# *Schizosaccharomyces pombe pus1* mutants are temperature sensitive due to decay of tRNA^Ile(UAU)^ by the 5′-3′ exonuclease Dhp1, primarily targeting the unspliced pre-tRNA

**DOI:** 10.1261/rna.080315.124

**Published:** 2025-04

**Authors:** Franziska Stegemann, Erin Marcus, Savanah Neupert, Sarah Ostrowski, David H. Mathews, Eric M. Phizicky

**Affiliations:** 1 Department of Biochemistry and Biophysics, Center for RNA Biology, University of Rochester School of Medicine, Rochester, New York 14642, USA

**Keywords:** rapid tRNA decay, Dhp1/Rat1, Tol1/Met22, tRNA^Ile(UAU)^, *S. pombe*

## Abstract

The pseudouridylase Pus1 catalyzes pseudouridine (Ψ) formation at multiple uridine residues in tRNAs, and in some snRNAs and mRNAs. Although Pus1 is highly conserved, and mutations are associated with human disease, little is known about eukaryotic Pus1 biology. Here, we show that *Schizosaccharomyces pombe pus1*Δ mutants are temperature sensitive due to decay of tRNA^Ile(UAU)^, as tRNA^Ile(UAU)^ levels are reduced, and its overexpression suppresses the defect. We show that tRNA^Ile(UAU)^ is degraded by the 5′-3′ exonuclease Dhp1 (ortholog of *Saccharomyces cerevisiae* Rat1), as each of four spontaneous *pus1*Δ suppressors had *dhp1* mutations and restored tRNA^Ile(UAU)^ levels, and two suppressors that also restored tRNA^Ile(UAU)^ levels had mutations in *tol1* (*S. cerevisiae MET22* ortholog), predicted to inhibit Dhp1. We show that Pus1 modifies U_27_, U_34_, and U_36_ of tRNA^Ile(UAU)^, raising the question about how these modifications prevent decay. Our results suggest that Dhp1 targets unspliced pre-tRNA^Ile(UAU)^, as a *pus1*Δ strain in which the only copy of tRNA^Ile(UAU)^ has no intron [*tI(UAU)-i*Δ] is temperature resistant and undergoes no detectable decay, and the corresponding *pus1*Δ *tI(UAU)-WT* strain accumulates unspliced pre-tRNA^Ile(UAU)^. Moreover, the predicted exon–intron structure of pre-tRNA^Ile(UAU)^ differs from the canonical bulge–helix–loop structure compatible with tRNA splicing, and a *pus1*Δ *tI(UAU)i-var* strain with intron mutations predicted to improve exon–intron structure is temperature resistant and undergoes little decay. These results suggest that decay of tRNA^Ile(UAU)^ by Dhp1 in *pus1*Δ strains occurs at the level of unspliced pre-tRNA^Ile(UAU)^, implying a substantial role for one or more of the Ψ residues in stabilizing the pre-tRNA structure for splicing.

## INTRODUCTION

The critical functions of tRNA in translation are significantly modulated by their numerous posttranscriptional modifications (Jackman and Alfonzo 2013; Boccaletto et al. 2018). Modifications are found in tRNAs in all organisms that have been examined, including the simplest (de Crecy-Lagard et al. 2012), and are highly conserved in different organisms, underscoring their importance (Jackman and Alfonzo 2013). Consistent with their importance, a lack of any of a number of modifications frequently results in growth or other phenotypic defects in the budding yeast *Saccharomyces cerevisiae* (Hopper 2013; Phizicky and Hopper 2023) and in neurological or other disorders in humans (Ramos and Fu 2019; Suzuki 2021; Phizicky and Hopper 2023).

Modifications have different types of roles depending on their location. Modifications in the anticodon loop (ACL) (residues N_31_–N_39_, including the closing base pair) play a number of important roles in promoting translation efficiency and/or fidelity (Urbonavicius et al. 2001; Nedialkova and Leidel 2015; Grosjean and Westhof 2016; Ranjan and Rodnina 2017; Phizicky and Hopper 2023). In contrast, modifications within the main tRNA body (N_1_–N_30_ and N_40_–N_73_) are generally important for folding and/or stability (Helm et al. 1999; Kadaba et al. 2004; Alexandrov et al. 2006; Chernyakov et al. 2008; De Zoysa and Phizicky 2020), although body modifications can also affect translation accuracy (Saleh and Farabaugh 2023).

In *S. cerevisiae*, lack of any of several different body modifications leads to reduced tRNA stability in vivo, resulting in decay of a subset of the hypomodified tRNAs by one or more of three mechanisms, depending on the particular modification (Phizicky and Hopper 2023). First, the rapid tRNA decay (RTD) pathway targets tRNAs for 5′-3′ exonucleolytic decay by Rat1 and Xrn1 in mutants lacking 7-methylguanosine at G_46_ (m^7^G_46_), *N*_2_,*N*_2_-dimethylguanosine at G_26_ (m^2,2^G_26_), or 4-acetylcytidine at C_12_ (ac^4^C_12_) in their tRNAs, particularly at higher temperatures, or in combination with a lack of other tRNA body modifications (Alexandrov et al. 2006; Chernyakov et al. 2008; Dewe et al. 2012; De Zoysa et al. 2024). The RTD pathway is inhibited by a *met22*Δ mutation (Chernyakov et al. 2008) due to the accumulation of the Met22 substrate adenosine 3′,5′-bisphosphate (pAp), which binds at the active site of the exonucleases (Dichtl et al. 1997; Yun et al. 2018). Second, the nuclear surveillance pathway targets pre-tRNA_i_^Met^ lacking 1-methyladenosine at A_58_ (m^1^A_58_) using the TRAMP complex to oligoadenylate the 3′ trailer, followed by 3′-5′ exonucleolytic decay by Rrp6 and Rrp44 of the nuclear exosome (Kadaba et al. 2004, 2006; Wang et al. 2008). Intriguingly, tRNA_i_^Met^ lacking m^1^A_58_ is also a substrate of the RTD pathway, and both pathways act in *S. cerevisiae* with comparable efficiency to maintain tRNA_i_^Met^ levels (Tasak and Phizicky 2022). Third, an as yet uncharacterized decay pathway requiring Met22 targets tRNA^Trp(CCA)^ lacking 1-methylguanosine at G_9_ (m^1^G_9_) for decay under normal growth conditions and in the presence of 5-fluorouracil (5-FU); however, unlike the RTD pathway, this pathway does not require the exonucleases Rat1 or Xrn1 (Bowles and Jackman 2024).

Similar analysis of body modification mutants in the evolutionarily distant fission yeast *Schizosaccharomyces pombe* has shown that the RTD pathway is also acting on a specific subset of hypomodified tRNA species in mutants lacking m^7^G_46_, m^1^A_58_, or ac^4^C_12_ (De Zoysa and Phizicky 2020; Tasak and Phizicky 2022; De Zoysa et al. 2024), establishing that the RTD pathway is conserved across at least the 600 million years separating *S. pombe* and *S. cerevisiae* (Parfrey et al. 2011).

Relatively little is known in any other eukaryote about the in vivo effect of lack of body modifications on tRNA folding, stability, or decay pathways. It has been known for some time that lack of m^1^A_9_ in human mitochondrial tRNA^Lys(UUU)^ results in altered folding (Helm et al. 1999; Helm and Attardi 2004), but the consequences in vivo are not known. In mouse cytoplasmic tRNAs, lack of m^5^C due to mutation of both NSUN2 and DNMT2 leads to reduced levels of tRNAs that are modified by both enzymes (Tuorto et al. 2012; Hussain et al. 2013). In addition, lack of m^7^G_46_ in mouse cells results in reduced levels of six of the 22 tRNAs with the modification, along with multiple cell gene expression and lineage-specific phenotypes ascribed to one of these tRNAs (Lin et al. 2018; Dai et al. 2021; Orellana et al. 2021). However, in both of these cases, it is not clear how the reduced tRNA levels are achieved.

Other than the relatively well-studied modifications described above (m^1^G_9_, ac^4^C_12_, m^2,2^G_26_, m^7^G_46_, m^1^A_58_), little is known in detail about other body modifications that might affect the folding and/or stability of tRNAs in any eukaryote, or mechanisms controlling their decay. Indeed, in *S. cerevisiae*, the lack of any of the 14 other body modifications (or sets of modifications catalyzed by one enzyme), has not been associated with any tRNA folding and/or stability defects, and with few exceptions (for example, Astrom et al. 1999), relatively little is known about their roles in *any* eukaryote.

We focus here on the biology of the pseudouridylase Pus1 in *S. pombe*. Pus1 is of interest for two reasons. First, Pus1 is highly conserved in eukaryotes and is highly promiscuous, modifying multiple uridine residues in different tRNAs, as well as uridine residues in several other classes of RNAs. In *S. cerevisiae*, Pus1 pseudouridylates tRNAs at U_1_, U_26_, U_27_, U_28_, U_34_, U_36_, U_65_, and U_67_ (Simos et al. 1996; Motorin et al. 1998; Behm-Ansmant et al. 2006), with recognition for modification at U_34_ and U_36_ of tRNA^Ile(UAU)^ requiring the intron-containing pre-tRNA (Szweykowska-Kulinska et al. 1994; Simos et al. 1996). In addition, *S. cerevisiae* Pus1 modifies U2 snRNA at U_44_ (Massenet et al. 1998), U6 snRNA at U_28_ under certain growth conditions (Basak and Query 2014), and multiple mRNAs at different specific uridine residues (Carlile et al. 2014; Schwartz et al. 2014). Similarly, *S. pombe* Pus1 modifies *S. cerevisiae* tRNA substrates in vitro at U_27_, U_28_, and U_35_, and pre-tRNA^Ile(UAU)^ at U_34_ and U_36_ (Hellmuth et al. 2000); human and mouse Pus1 each pseudouridylate similar substrate tRNAs at U_27_ and U_28_ and pre-tRNA^Ile(UAU)^ at U_34_ and U_36_ (Chen and Patton 1999; Sibert et al. 2008); mouse Pus1 additionally modifies tRNAs at U_1_ (Behm-Ansmant et al. 2006) and the steroid receptor RNA activator (Zhao et al. 2004); and human Pus1 also modifies mRNAs (Li et al. 2015). This promiscuity of Pus1 is due in part to the lack of a strongly defined structural motif and sequence recognition logo (Sibert and Patton 2012; Carlile et al. 2019), and together with its conservation, makes it all the more important to uncover Pus1 roles in eukaryotes.

Second, understanding the biology of Pus1 could shed light on the biology of human *PUS1*, mutation of which has been associated with the rare autosomal recessive disease mitochondrial myopathy and sideroblastic anemia (MLASA) and the related condition congenital sideroblastic anemia (CSA) (Bykhovskaya et al. 2004; Fernandez-Vizarra et al. 2007; Bergmann et al. 2010; Fujiwara and Harigae 2013).

In addition, there is some indication that Pus1 biology might be amenable to study in fungi. *S. cerevisiae pus1*Δ mutants are known to be modestly temperature sensitive (Gustavsson and Ronne 2008; Li et al. 2009; Khonsari and Klassen 2020), and this temperature sensitivity is modestly suppressed by a *met22*Δ mutation, suggesting that some tRNAs may be subject to RTD (Khonsari and Klassen 2020), or another Met22-dependent tRNA decay pathway (Bowles and Jackman 2024).

Here, we show that *S. pombe pus1*Δ mutants are temperature sensitive due primarily to decay of tRNA^Ile(UAU)^ by the RTD pathway. In support of this, we find that *pus1*Δ temperature sensitivity is associated with reduced levels of tRNA^Ile(UAU)^ (but not each of 16 other tRNAs), that overexpression of tRNA^Ile(UAU)^ almost completely suppresses the temperature sensitivity, and that several spontaneous suppressors of *pus1*Δ temperature sensitivity have mutations in the *RAT1* ortholog *dhp1* or the *MET22* ortholog *tol1* and substantially restore tRNA^Ile(UAU)^ levels. In addition, we find that another class of *S. pombe pus1*Δ suppressors have mutations in ribosomal protein subunits, as we recently reported for suppressors of *S. pombe* mutants lacking m^7^G_46_ or ac^4^C_12_ in their tRNAs (De Zoysa et al. 2024).

Our further analysis shows that Pus1 normally pseudouridylates *S. pombe* tRNA^Ile(UAU)^ at U_27_, U_34_, and U_36_ in vivo and provides evidence that decay occurs in a *pus1*Δ strain at the level of unspliced pre-tRNA^Ile(UAU)^. In support of this, we show that the temperature sensitivity of a *pus1*Δ strain with a single WT *tI(UAU)* gene [*pus1*Δ *tI(UAU)-WT*] is accompanied by the accumulation of unspliced pre-tRNA^Ile(UAU)^, whereas a *pus1*Δ strain with a single *tI(UAU)* lacking an intron (*pus1*Δ *tI(UAU)-i*Δ) grows well at elevated temperature and does not undergo decay of tRNA^Ile(UAU)^. Our analysis shows that the predicted exon–intron structure of the pre-tRNA^Ile(UAU)^ lacks the canonical bulge–helix–loop (BHL) or bulge–helix–bulge (BHB) that is compatible with efficient tRNA splicing (Baldi et al. 1992; Schmidt and Matera 2020; Hayne et al. 2023a,b; Sekulovski et al. 2023), suggesting a link between inefficient splicing and pre-tRNA^Ile(UAU)^ decay. Consistent with this, we find that each of three *pus1*Δ *tI(UAU)i-var* strains with intron mutations predicted to improve exon–intron structure grows well at elevated temperature and, for the one that was tested, has little obvious decay of tRNA^Ile(UAU)^, and does not accumulate unspliced pre-tRNA^Ile(UAU)^. We interpret these results in terms of a model in which an *S. pombe pus1*Δ strain is temperature sensitive due to decay of unspliced pre-tRNA^Ile(UAU)^ that accumulates at elevated temperature and, based on the increased accumulation of pre-tRNA in a *pus1*Δ strain, suggest that Ψ_34_ and Ψ_36_ alter the exon–intron structure to favor splicing, in addition to their presumed roles in decoding in the ribosome A site.

Our findings also extend the spectrum of body modification mutants in *S. pombe* that are subject to decay by the RTD pathway or related decay pathways, leading to speculation that other body modification mutants in these organisms and perhaps metazoans will also trigger tRNA decay by similar pathways.

## RESULTS

### *S. pombe pus1*Δ mutants are temperature sensitive and have the anticipated reduced levels of pseudouridine

**FIGURE 1.**
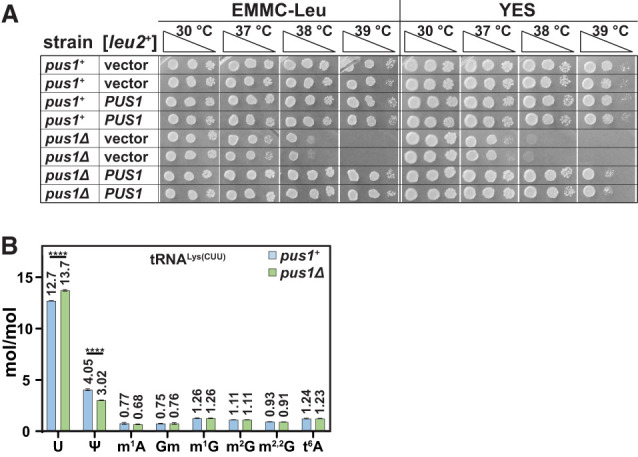
*S. pombe pus1Δ* strains are temperature sensitive and lack Ψ at anticipated sites in tRNAs. (*A*) *S. pombe pus1*Δ strains are temperature sensitive and are complemented by overexpression of *pus1*^*+*^. Independent [*pus1*^*+*^ *leu2*^*+*^] or [*leu2*^*+*^] transformants of WT and *pus1*Δ cells were grown overnight in EMMC-Leu media at 30°C. Then cells were diluted to an OD_600_ of 0.5, serially diluted 10-fold, and spotted for growth analysis at the indicated temperatures on EMMC-Leu and YES media for 4 days. (*B*) tRNA^Lys(CUU)^ purified from *S. pombe pus1*Δ strains has reduced Ψ and commensurately increased uridine, relative to that from WT strains. WT and *pus1*Δ strains were grown in biological triplicates, and tRNA^Lys(CUU)^ was purified as described in Materials and Methods. Then RNA was digested to nucleosides, which were analyzed by HPLC as described in Materials and Methods. Levels of nucleosides in WT and *pus1*Δ strains are shown in blue and green, respectively. Standard deviations are indicated. The statistical significance was evaluated using a one-tailed Student's *t*-test assuming equal variance, indicated by (****) for *P* < 0.0001.

To study the biology of *S. pombe* Pus1, we used a hygromycin resistance cassette to generate *pus1*Δ mutants, and then analyzed growth. We found that each of the three independent *pus1*Δ mutants grows similarly to wild-type (WT) cells at 30°C, but is temperature sensitive on both rich (YES) media and minimal (EMMC-His) media, starting at 37°C (Supplemental Fig. S1A). As the temperature sensitivity of a representative *pus1*Δ strain is complemented by the expression of *pus1*^*+*^ from a [*leu2*^*+*^ *pus1*^*+*^] plasmid, but not by the vector control (Fig. 1A), we conclude that the *pus1*Δ mutation is responsible for the temperature sensitivity.

To begin to assess the effects of the *pus1*Δ mutation on Ψ modification, we compared WT and *pus1*Δ strains for the Ψ content of the population of 37 isodecoder tRNA families lacking a long variable loop (Chan and Lowe 2016), by HPLC analysis of nucleosides (see Materials and Methods). Relative to the amount of cytidine, we find that the *pus1*Δ mutants have significantly reduced Ψ levels (13.3%), compared to levels in the WT strain (16.7%), and significantly more uridine than the WT strain (72.8% vs. 69.9%), whereas levels of each of six other modifications are very similar, as are levels of adenosine and guanosine (Supplemental Fig. S1B). This distinct, but modest, reduction in overall Ψ modification in *S. pombe pus1*Δ mutants is consistent with the modest fraction of total Ψ modification in characterized *S. cerevisiae* tRNAs that is due to Pus1 (Simos et al. 1996; Motorin et al. 1998; Behm-Ansmant et al. 2006; Juhling et al. 2009; Boccaletto et al. 2018), and the similar specificity of the *S. cerevisiae* and *S. pombe* Pus1 proteins (Hellmuth et al. 2000).

To further evaluate the loss of pseudouridylation in *S. pombe pus1*Δ mutants, we purified and analyzed tRNA^Lys(CUU)^, which, based on its sequence, was expected to have a Pus1-dependent Ψ modification at U_27_, as is found in the corresponding *S. cerevisiae* tRNA. Consistent with this expectation, we found that purified tRNA^Lys(CUU)^ from *pus1*Δ mutants has 1.03 fewer moles/mole of Ψ than that from a WT strain (3.02 vs. 4.05 moles/mole), with a corresponding increase of 1.0 mole/mole of uridine (13.7 vs. 12.7 moles/mole), whereas each of six other modifications is present at almost identical levels in both *pus1*Δ and WT strains (Fig. 1B). These results demonstrate that *S. pombe* Pus1 pseudouridylates tRNAs in vivo and that tRNA^Lys(CUU)^ is one of its substrates.

### *S. pombe pus1*Δ temperature sensitivity is due to reduced levels of tRNA^Ile(UAU)^

To determine if the temperature sensitivity of *S. pombe pus1*Δ strains is associated with reduced levels of tRNA species, we examined a number of tRNAs in WT and *pus1*Δ strains during log phase growth at 30°C, and at intervals up to 6 h after a temperature shift to 38.5°C. The *pus1*Δ strains grew significantly more poorly in liquid YES media at 38.5°C, beginning 1 h after the temperature shift (Supplemental Fig. S2A), and plating experiments show that after the temperature shift cell viability was unaffected (Supplemental Fig. S2B). As described previously (De Zoysa and Phizicky 2020), we measured relative levels of each tRNA species in each strain at each temperature, by normalization of the hybridization signal of that tRNA species to that of tRNA^Gly(GCC)^ (which does not change) for the corresponding strain and temperature, followed by renormalization to the levels of that tRNA in the WT strains at 30°C.

**FIGURE 2.**
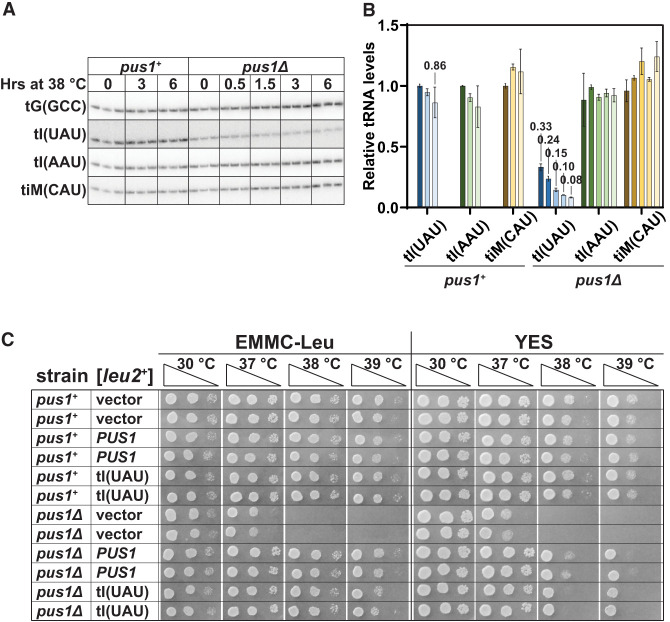
*S. pombe pus1*Δ strains are temperature sensitive due to reduced levels of tRNA^Ile(UAU)^. (*A*) *S. pombe pus1*Δ strains have reduced levels of tRNA^Ile(UAU)^ at 30°C, which are further reduced at 38.5°C, relative to levels in WT cells. *S. pombe* WT and *pus1*Δ strains were grown in biological triplicate in YES media at 30°C and shifted to 38.5°C. Bulk RNA was prepared from cells at time points as indicated and analyzed by northern blotting with probes as shown. (*B*) Quantification of tRNA levels in *S. pombe* WT and *pus1*Δ strains at 30°C and 38.5°C. The bar chart shows relative levels of tRNA species, each normalized to levels of the control tRNA^Gly(GCC)^ followed by normalization to the normalized level in the WT strain at 30°C. Levels of tRNA^Ile(UAU)^, tRNA^Ile(AAU)^, and tRNA_i_^Met(CAU)^ are depicted in blue, green, and yellow, respectively, with dark shades at 30°C, and progressively lighter shades after 3 and 6 h at 38.5°C for WT cells, and after 0.5, 1.5, 3, and 6 h for *pus1*Δ mutants, as indicated. Standard deviations are shown. (*C*) Overexpression of tRNA^Ile(UAU)^ suppresses the temperature-sensitive growth defects of *S. pombe pus1*Δ strains. WT and *pus1*Δ cells were transformed with [*leu2*^*+*^], [*pus1*^*+*^ *leu2*^*+*^], or [*tI(UAU) leu2*^*+*^] as indicated. Independent transformants were grown overnight in EMMC-Leu media at 30°C, and cells were diluted and spotted as described in Figure 1A on EMMC-Leu and YES media, and incubated for 3 days at the indicated temperatures.

Using this approach, we find that relative tRNA^Ile(UAU)^ levels are significantly reduced in *pus1*Δ strains at 30°C (33% of WT), and are further reduced during incubation at 38.5°C, to 8% after 6 h, whereas relative levels of tRNA^Ile(UAU)^ are only marginally reduced in WT strains after 6 h at 38.5°C (86%) (Fig. 2A,B). In contrast, relative levels of two control tRNAs, tRNA^Ile(AAU)^_,_ and tRNA_i_^Met(CAU)^, remain essentially at WT levels in the *pus1*Δ mutant at both 30°C and 38.5°C (Fig. 2A,B), as do each of 14 other tRNAs examined (Supplemental Figs. S2C, S3A,B), with relative tRNA levels ranging between 68% and 140% in *pus1*Δ strains, and between 76% and 120% in WT strains.

To determine if the temperature sensitivity of *S. pombe pus1*Δ mutants is due to the loss of tRNA^Ile(UAU)^, we analyzed the growth of *pus1*Δ strains at high temperatures after overexpression of tRNA^Ile(UAU)^ on a [*leu2^+^ tI(UAU)*] plasmid. We find that the temperature sensitivity of *S. pombe pus1*Δ strains is virtually completely suppressed by tRNA^Ile(UAU)^ overexpression, as these strains grow almost as well on EMMC-Leu media at both 38°C and 39°C as control *pus1*Δ [*leu2*^*+*^ *pus1*^*+*^] and WT strains (Fig. 2C), and nearly as well as the *pus1*Δ [*leu2*^*+*^ *pus1*^*+*^] strains on YES media (on which plasmid loss can occur). As expected, Northern analysis of these strains shows that tRNA^Ile(UAU)^ levels are overexpressed in *pus1*Δ [*leu2^+^ tI(UAU)*] strains compared to a WT strain, by 6.5-fold at 30.0°C, and by 3.5-fold after 6 h at 38.5°C (Supplemental Fig. S4A,B), whereas levels of two other tRNAs (tRNA^Ala(AGC)^ and tRNA^Cys(GCA)^) remain almost constant. These results show that the temperature sensitivity of *S. pombe pus1*Δ strains is caused by loss of tRNA^Ile(UAU)^ and therefore that tRNA^Ile(UAU)^ is the primary biologically relevant Pus1 tRNA substrate under these growth conditions.

### Spontaneous suppressors of the temperature sensitivity of *S. pombe pus1*Δ strains have mutations in components of the RTD pathway and restore tRNA^Ile(UAU)^ levels

**FIGURE 3.**
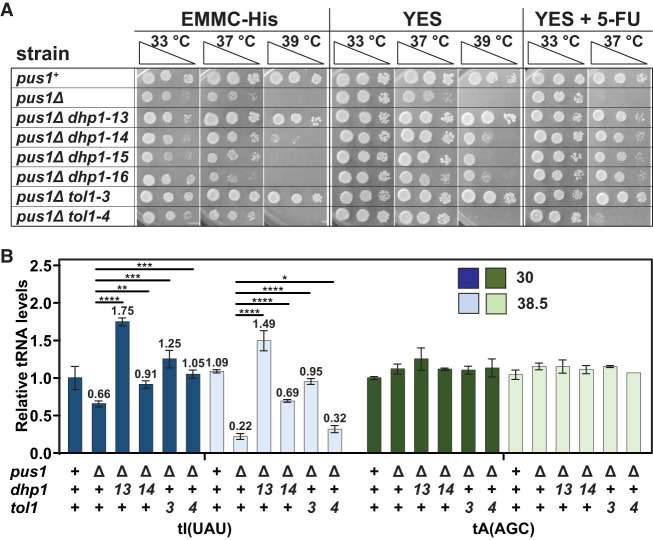
Spontaneous *pus1*Δ suppressors carrying mutations in genes of the RTD pathway lead to restoration of growth and tRNA^Ile(UAU)^ levels in *S. pombe pus1*Δ strains. (*A*) Spontaneous suppressors carrying mutations in *dhp1* or *tol1* suppress the temperature-sensitive phenotype of *S. pombe pus1*Δ strains. Strains were cultivated in YES media at 30°C, serially diluted, and analyzed for growth on EMMC-His, YES, and YES + 5-FU media. Cells on EMMC-His are shown after 4 days of incubation, cells on YES and YES + 5-FU are shown after 6 days of incubation. (*B*) Spontaneous *pus1*Δ suppressors carrying mutations in *dhp1* or *tol1* restore tRNA^Ile(UAU)^ levels in *S. pombe pus1*Δ strains at 30°C and 38.5°C. Strains were grown in biological replicates in YES media at 30°C and shifted to 38.5°C for 6 h, and then RNA was isolated and analyzed by Northern blotting. The bar chart shows relative levels of tRNA species. Strains are analyzed at 30°C (dark shades) and 38.5°C (light shades). Levels of tRNA^Ile(UAU)^ and tRNA^Ala(AGC)^ are depicted in blue and green, respectively. Standard deviations are indicated. The statistical significance was evaluated using a one-tailed Student's *t*-test assuming equal variance, indicated by (*) for *P* < 0.05, (**) for *P* < 0.01, (***) for *P* < 0.001, (****) for *P* < 0.0001.

To identify biological pathways that are responsible for the growth defect of *S. pombe pus1*Δ strains, we isolated and analyzed spontaneous suppressors of the temperature-sensitive phenotype. Among 28 *pus1*Δ suppressors analyzed by whole-genome sequencing, we identified four different alleles of the *RAT1* ortholog *dhp1*, and two different alleles of the *MET22* ortholog *tol1*. All of the *pus1*Δ *dhp1* and *pus1*Δ *tol1* suppressors restore growth on YES media and/or on EMMC-His media at elevated temperatures, with the strongest suppression by the *pus1*Δ *dhp1*-*13* and *pus1*Δ *tol1-3* strains, and the weakest suppression by the *pus1*Δ *tol1-4* strain (Fig. 3A). In addition, all of the suppressor strains except for the very weak *pus1*Δ *tol1-4* suppressor strain also confer resistance on YES media containing 5-fluorouracil (5-FU) at 37°C, compared to that of the *pus1*Δ strain. We had previously found that *trm8*Δ *dhp1* and *tan1*Δ *dhp1* suppressor strains were resistant to 5-FU, which we ascribed to the effect of 5-FU on inhibiting certain Ψ and methyl modifications (Frendewey et al. 1982; Santi and Hardy 1987; Huang et al. 1998), and the enhanced protection from decay afforded by the *dhp1* mutations in *trm8*Δ and *tan1*Δ strains after treatment with 5-FU (De Zoysa and Phizicky 2020; De Zoysa et al. 2024).

These *dhp1* and *tol1* alleles are almost certainly responsible for the suppression of the *S. pombe pus1*Δ temperature sensitivity, for two reasons. First, the finding of multiple different alleles of any particular gene among genetically independent suppressors is highly unlikely to occur by chance, as only a few mutations are typically detected by whole-genome sequencing of any particular spontaneous suppressor strain, and the chance occurrence of different alleles of the *dhp1* and *tol1* genes is particularly unlikely as they are each essential (Sugano et al. 1994; Miyamoto et al. 2000). Second, explicit testing of one of the *dhp1* and *tol1* mutations shows that the mutation is responsible for suppression of the *S. pombe pus1*Δ temperature sensitivity. The strongest *pus1*Δ *dhp1* suppressor (*dhp1-13*) carries the S737P mutation, which we also previously found as an *S. pombe trm6*Δ suppressor (Tasak and Phizicky 2022), and we find that a reconstructed *pus1*Δ *dhp1*-*S737P* strain is also strongly temperature resistant (Supplemental Fig. S5A). Similarly, we find that introduction of a [*leu2*^*+*^ *tol1*^*+*^] plasmid complements the temperature resistance of the strong *pus1*Δ *tol1-3* suppressor, resulting in temperature sensitivity that is similar to that of the parent *pus1*Δ strain on EMMC-Leu media (Supplemental Fig. S5B). These results strongly suggest that the four *dhp1* and two *tol1* alleles are each responsible for suppression of the temperature sensitivity in the corresponding *pus1*Δ suppressor strains.

Northern analysis shows that tRNA^Ile(UAU)^ levels are significantly restored at low and high temperature in each of two tested *pus1*Δ *dhp1* suppressor strains and in both of the *pus1*Δ *tol1* suppressor strains. At 38.5°C, relative tRNA^Ile(UAU)^ levels increase from 22% in *pus1*Δ strains to 149%, 69%, 95%, and 32% in the *pus1*Δ *dhp1-13*, *pus1*Δ *dhp1-14*, *pus1*Δ *tol1-3,* and *pus1*Δ *tol1-4* mutants, respectively (Fig. 3B; Supplemental Fig. S6A). Similarly, at 30°C, relative tRNA^Ile(UAU)^ levels increase from 65% in *pus1*Δ strains to 175%, 91%, 125%, and 105% in the *pus1*Δ *dhp1-13*, *pus1*Δ *dhp1-14*, *pus1*Δ *tol1-3,* and *pus1*Δ *tol1-4* mutants, respectively. In contrast, the relative levels of the controls tRNA^Ala(AGC)^ and tRNA^Cys(GCA)^ (Fig. 3B; Supplemental Fig. S6B) are almost completely constant in each strain and at each temperature.

These results demonstrate that the temperature sensitivity of the *S. pombe pus1*Δ strains is due to decay of tRNA^Ile(UAU)^ by Dhp1/Rat1 and Tol1/Met22 of the RTD pathway. Moreover, as the efficiency of restoration of tRNA^Ile(UAU)^ levels in the *pus1*Δ *dhp1* and *pus1*Δ *tol1* suppressors (Fig. 3B) corresponds to the efficiency of suppression of *pus1*Δ temperature sensitivity (Fig. 3A), we infer that growth in a *pus1*Δ strain at high temperature is limited by the levels of tRNA^Ile(UAU)^. In addition, our finding that tRNA^Ile(UAU)^ levels are also increased at 30°C in the *pus1*Δ *dhp1* and *pus1*Δ *tol1* suppressors documents that the RTD pathway is also acting on tRNA^Ile(UAU)^ at low temperature in *S. pombe pus1*Δ mutants.

Control experiments also suggest that the nuclear surveillance pathway has a minimal role in degrading tRNA^Ile(UAU)^ in an *S. pombe pus1*Δ strain, as disruption of the *TRF4* ortholog *cid14*^*+*^ in a *pus1*Δ strain results in very weak growth suppression on EMMC-His and YES media at 37°C, but not at higher temperatures (Supplemental Fig. S7).

### Several suppressors of the *S. pombe pus1*Δ growth defect have mutations predicted to affect ribosome levels

Among the 28 spontaneous *pus1*Δ suppressors analyzed by whole-genome sequencing, we also identified six with mutations in genes affecting the ribosome (Supplemental Fig. S8). We obtained three *pus1*Δ suppressors with *rpl* mutations, encoding proteins of the large subunit (*rpl1101-N7fs*, *rpl502-R23-R24del*, and *rpl1001-F119fs*, encoding uL5, uL18, and uL16, respectively), and one with an *rps* mutation, encoding a protein of the small subunit (*rps1901-P31del*, encoding eS19). In addition, we found two *pus1*Δ suppressors with mutations in *dbp7* (*dbp7-L193X* and *dbp7-R388X*), encoding an ATP-dependent RNA helicase which, in *S. cerevisiae*, is important for 60S ribosome biogenesis (Daugeron and Linder 1998).

These results are consistent with our previous observations that mutations in ribosomal protein genes frequently arise among suppressors of the temperature sensitivity of *S. pombe trm8*Δ mutants and *tan1*Δ mutants, each of which is temperature sensitive due to RTD (De Zoysa et al. 2024). Indeed, as we showed previously for *trm8*Δ *rp* mutants, *tan1*Δ *rp* mutants, and otherwise WT *rp* mutant strains (De Zoysa et al. 2024), each of these *pus1*Δ *rp* suppressors is more resistant to 3-aminotriazole, an activator of the general amino acid control pathway, than control WT, *pus1*Δ, *pus1*Δ *dhp1* suppressors, or *pus1*Δ *tol1* suppressors, albeit to different extents (Supplemental Fig. S8).

### *S. pombe* tRNA^Ile(UAU)^ has three Ψ modification sites directed by Pus1

**FIGURE 4.**
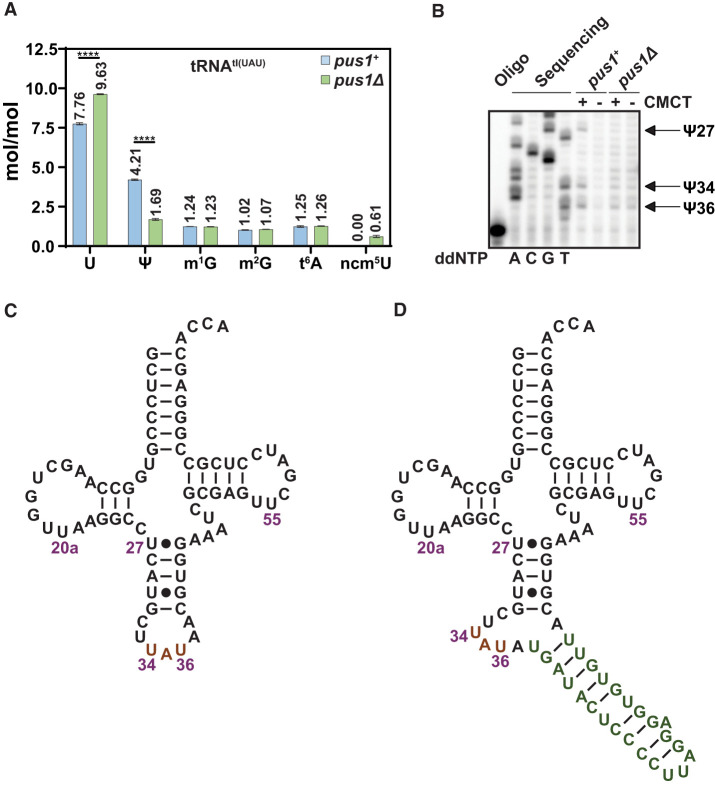
*S. pombe* Pus1 pseudouridylates tRNA^Ile(UAU)^ at U_27_, U_34_, and U_36_. (*A*) tRNA^Ile(UAU)^ purified from *S. pombe pus1*Δ strains has reduced Ψ and commensurately increased uridine and ncm^5^U, relative to WT strains. WT and *pus1*Δ strains were transformed with a [*leu2^+^ tI(UAU)*] plasmid, and transformants were grown in triplicate in EMMC-Leu media at 30°C to mid-log phase, and harvested. Then tRNA^Ile(UAU)^ was purified from bulk RNA and digested to nucleosides, which were analyzed by HPLC. Levels of nucleosides in WT and *pus1*Δ strains are shown in blue and green, respectively. Standard deviations are indicated. The statistical significance was evaluated using a one-tailed Student's *t*-test assuming equal variance, indicated by (****) for *P* < 0.0001. (*B*) tRNA^Ile(UAU)^ purified from *S. pombe pus1*Δ strains lacks Ψ_27_, Ψ_34_, and Ψ_36_, compared to WT strains. Purified tRNA was treated with CMCT and analyzed by primer extension, as described in Materials and Methods, with primer OFS049 (complementary to tRNA^Ile(UAU)^ nt 57–39). The primer extension stops in tRNA^Ile(UAU)^ from WT cells occur one residue 3′ of Ψ_27_, Ψ_34_, and Ψ_36_, but are absent in tRNA^Ile(UAU)^ from *pus1*Δ strains, demonstrating the lack of Ψ_27_, Ψ_34_, and Ψ_36_ in tRNA^Ile(UAU)^ from *pus1*Δ strains. A sequencing ladder is shown on the *left*. (*C*) Secondary structure of mature *S. pombe* tRNA^Ile(UAU)^. The anticodon is orange. (*D*) Secondary structure of intron-containing *S. pombe* tRNA^Ile(UAU)^. The anticodon is highlighted in orange, the intron is green.

To assess the Pus1 Ψ modifications that prevent decay of *S. pombe* tRNA^Ile(UAU)^ in WT cells, we compared the Ψ content of tRNA^Ile(UAU)^ purified from WT and *pus1*Δ mutants. As *S. pombe* has only a single gene encoding tRNA^Ile(UAU)^, we used strains transformed with a [*leu2^+^ tI(UAU)*] plasmid, in which tRNA^Ile(UAU)^ is overexpressed (Supplemental Fig. S4). We find that tRNA^Ile(UAU)^ from a *pus1*Δ strain has 2.52 fewer moles Ψ/mole tRNA than from a WT strain (1.69 vs. moles 4.21 Ψ/mole) (Fig. 4A), suggesting three sites for Pus1 pseudouridylation, compensated by an additional 1.87 moles/mole of uridine (9.63 vs. 7.76 moles/mole in WT), and an additional 0.61 moles/mole of ncm^5^U in the *pus1*Δ strain, which is not detectable in WT. In contrast, the levels of each of the three other modifications (m^1^G, m^2^G, and t^6^A) are almost identical in the *pus1*Δ and WT strains. The additional ncm^5^U in the *pus1*Δ strain is undoubtedly at U_34_, consistent with the documented occurrence of ncm^5^U only at U_34_ (Huang et al. 2005; Karlsborn et al. 2014; Boccaletto et al. 2018), and with the known accumulation of ncm^5^U_34_ in submolar amounts in *S. cerevisiae* tRNA^Ile(UAU)^ in strains lacking its intron (Hayashi et al. 2019), which is required for Pus1 modification of U_34_ and U_36_ of tRNA^Ile(UAU)^ (Szweykowska-Kulinska et al. 1994; Motorin et al. 1998).

To map the Pus1 Ψ modification sites in tRNA^Ile(UAU)^, we identified the pseudouridylation sites in *S. pombe* WT and *pus1*Δ strains by mapping with N-cyclohexyl-N′-β-(4-methylmorpholinium) ethylcarbodiimide *p*-tosylate (CMCT) (Bakin and Ofengand 1993; Huang et al. 2016). After CMCT treatment, a primer extension of a 5′ ^32^P-labeled primer hybridizing from nt 57 to 39 reveals prominent stops in the WT but not the *pus1*Δ strain at C_28_, A_35_, and A_37_, indicating that *S. pombe* Pus1 modifies tRNA^Ile(UAU)^ at U_27_, U_34_, and U_36_ (Fig. 4B–D). This Pus1 modification pattern is consistent with the known Pus1 Ψ modification of *S. cerevisiae* tRNA^Ile(UAU)^ at these residues (Motorin et al. 1998), with Ψ_34_, and Ψ_36_ modification requiring the intron (Szweykowska-Kulinska et al. 1994; Simos et al. 1996; Hayashi et al. 2019), and with the analysis of *S. pombe* Pus1 catalytic activity with an *S. cerevisiae* tRNA^Ile(UAU)^ transcript (Hellmuth et al. 2000). As expected, CMCT mapping also reveals a Ψ modification site at U_55_ of *S. pombe* tRNA^Ile(UAU)^ (Supplemental Fig. S9), which is found in both WT and *pus1*Δ strains, as it is due to Pus4 (Becker et al. 1997).

These Pus1 Ψ mapping results are intriguing because it is not obvious why the lack of Ψ at U_27_, U_34_, and U_36_ in tRNA^Ile(UAU)^ triggers its decay by Dhp1/Rat1. Rat1 is a 5′-3′ exonuclease of the XRN family (Kenna et al. 1993; Stevens and Poole 1995; Xiang et al. 2009) which, in *S. cerevisiae*, acts with Xrn1 on substrate hypomodified mature tRNAs to catalyze 5′-3′ decay by the RTD pathway (Chernyakov et al. 2008; Whipple et al. 2011). However, it is difficult to rationalize how lack of Ψ_27_ could lead to 5′-3′ decay of tRNA^Ile(UAU)^ because N_27_ is in the anticodon stem, which does not participate in the overall tertiary fold of tRNA, and would not be expected to increase melting of the 5′ end of tRNA^Ile(UAU)^ for Dhp1 attack. Similarly, it is difficult to rationalize how lack of Ψ_34_ and Ψ_36_ in tRNA^Ile(UAU)^ would promote 5′-3 decay because the ACL does not interact with the main body of the tRNA, and lack of Ψ modification in the ACL would not increase melting of the 5′ end of tRNA^Ile(UAU)^ for Dhp1 attack.

### The temperature sensitivity of a *pus1*Δ strain is suppressed by removal of the intron of tRNA^Ile(UAU)^ or by a *tI(UAU)-U27C* mutation

**FIGURE 5.**
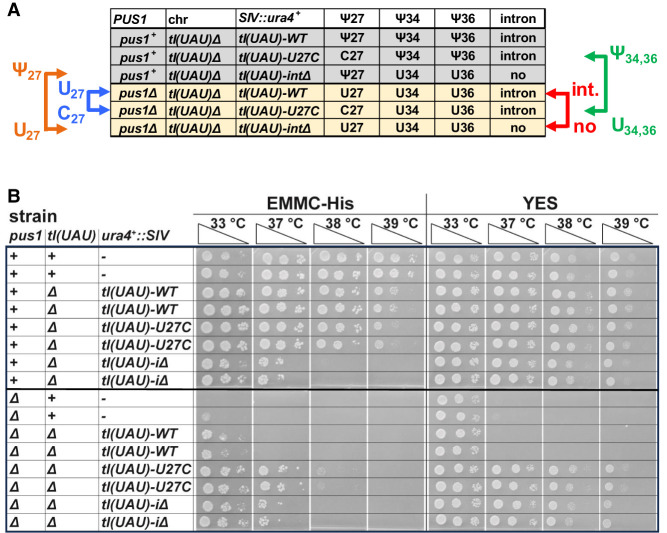
The temperature sensitivity of *pus1*Δ strains is suppressed if the single copy *tIUAU)* gene lacks its intron, or has a stabilizing U_27_C mutation. (*A*) Schematic showing the predicted residues at N_27_, N_34_, and N_36_ for *tIUAU)*-*WT*, *tIUAU)*-*U27C*, and *tIUAU)-i*Δ variants in WT and *pus1*Δ strains. Arrows point to explicit comparisons that can be made to assess the contributions of Ψ_27_ versus U_27_, U_27_ versus C_27_, Ψ_34_ and Ψ_36_ versus U_34_, and U_36_, and the intron versus no intron. (*B*) Growth properties of WT and *pus1*Δ strains with single integrated *tIUAU)* variants. The set of WT and *pus1*Δ strains depicted in *A* was constructed as described in the text. Then independent strains were grown overnight in YES media at 30°C, and then cells were serially diluted and spotted as described in Figure 1A on EMMC-Leu and YES media and incubated for 4 days and 2 days, respectively, at the indicated temperatures.

To test the importance of pseudouridine modification of U_27_, U_34_, and U_36_ in tRNA^Ile(UAU)^ function, we replaced the single copy essential *tI(UAU)* gene encoding tRNA^Ile(UAU)^ with *tI(UAU)* variants, in both WT and *pus1*Δ strains, and then tested the growth properties of the resulting strains (Fig. 5A). To accomplish this, we first introduced *tI(UAU)* variants at the *ura4* locus, using a stable integrating vector (SIV) (Vjestica et al. 2020) to generate strains with an additional *tI(UAU)* gene, and then deleted the original *tI(UAU)* gene at its normal chromosomal locus to generate strains with a single *tI(UAU)* variant at the *ura4* locus. In this way, we generated a set of WT and *pus1*Δ strains expressing *tI(UAU)-WT*; *a tI(UAU)-U_27_C* variant, replacing the weak U_27_:G_43_ base pair with the more stable C_27_:G_43_ pair; or a *tI(UAU)-i*Δ variant, lacking its intron (Fig. 4D), which is required for Ψ modification of both U_34_ and U_36_ (Szweykowska-Kulinska et al. 1994; Simos et al. 1996; Hayashi et al. 2019). Comparison of the growth properties of the resulting strains with *tI(UAU)* variants allows explicit comparison of the importance of the intron versus no intron; U_27_ versus C_27_; Ψ_34_, and Ψ_36_ versus U_34_, and U_36_; and U_27_ versus Ψ_27_ (Fig. 5A).

One major conclusion from this analysis is that the temperature sensitivity of a *pus1*Δ strain is efficiently suppressed by the removal of the intron or by a U_27_C mutation. Thus, both a *pus1*Δ *tI(UAU)-i*Δ strain and a *pus1*Δ *tI(UAU)-U27C* strain grow as well as the corresponding *pus1*^*+*^ strains on YES media at all temperatures, whereas the control *pus1*Δ *tI(UAU)-WT* strain is as temperature sensitive on YES media as the *pus1*Δ strain with the WT *tI(UAU)* gene at its native location (Fig. 5B). On minimal EMMC-His media at 37°C, we also observe suppression of the *pus1*Δ temperature sensitivity by removal of the *tI(UAU)* intron or by a U_27_C variant.

Our evidence also suggests that in the context of strains with *tI(UAU)* variants, modification of U_34_ and U_36_ to Ψ_34_ and Ψ_36_ is somewhat important for the function of tRNA^Ile(UAU)^, whereas modification of U_27_ to Ψ_27_ has a more minor role. As a *pus1^+^ tI(UAU)-U27C* strain grows well in EMMC-His media at 38°C, and modestly at 39°C, whereas the corresponding *pus1*Δ *tI(UAU)-U27C* strain grows well only at 37°C (Fig. 5A,B), we infer that Ψ_34_ and Ψ_36_ contribute somewhat to the function of the tRNA^Ile(UAU)^-U27C variant at higher temperature, compared to U_34_ and U_36_. Similarly, as a *pus1^+^ tI(UAU)-i*Δ strain grows very slightly better in EMMC-His media at 37°C than the corresponding *pus1*Δ *tI(UAU)i*Δ strain, we infer that Ψ_27_ has a slight, but decidedly minor, role in contributing to the function of the *tI(UAU)-i*Δ variant compared to U_27_.

Of additional interest, we note that a *pus1^+^ tI(UAU)-i*Δ strain is temperature sensitive on EMMC-His media and also to some extent on YES media, compared to the control *pus1^+^ tI(UAU)-WT* strain. This result suggests the possibility of a role for Ψ_34_ and Ψ_36_ in the function of tRNA^Ile(UAU)^, and is discussed further below.

### A *pus1*Δ *tI(UAU)-i*Δ strain and a *pus1*Δ *tI(UAU)-U27C* strain undergo little tRNA^Ile(UAU)^ decay, whereas a control *pus1*Δ *tI(UAU)-WT* strain accumulates unspliced pre-tRNA^Ile(UAU)^ and undergoes tRNA^Ile(UAU)^ decay

**FIGURE 6.**
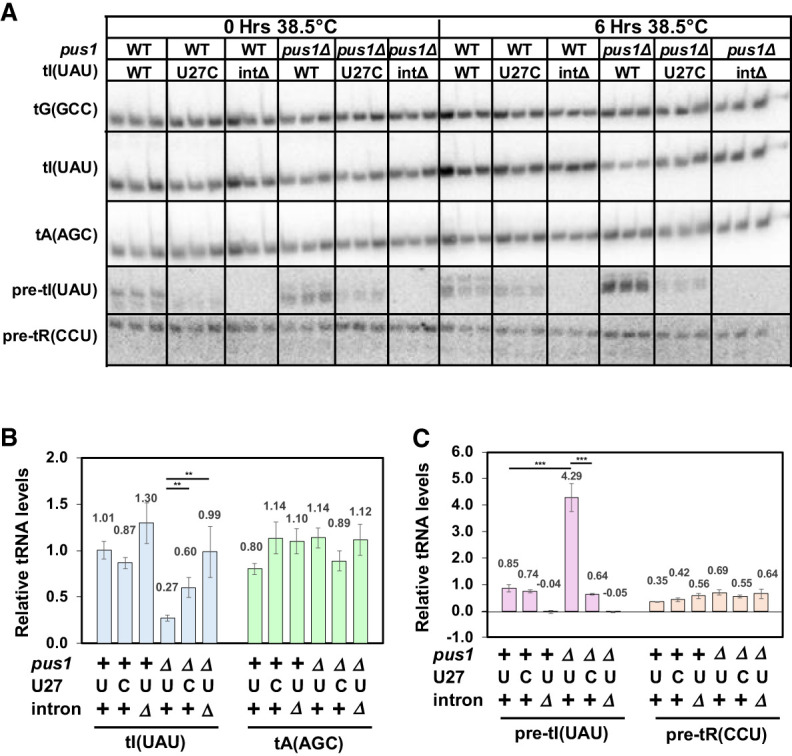
A *pus1*Δ *tI(UAU)-i*Δ strain and a *pus1*Δ *tI(UAU)-U27C* strain undergo little tRNA^Ile(UAU)^ decay, and the control *pus1*Δ *tI(UAU)-WT* strain accumulates unspliced pre-tRNA^Ile(UAU)^. (*A*) Northern analysis of *S. pombe* WT and *pus1*Δ strains with *tI(UAU)* variants. Strains as indicated were grown in biological replicates in YES media at 30°C and shifted to 38.5°C for 6 h, and then RNA was isolated and analyzed by Northern blotting, as in Figure 2A. (*B*) Quantification of levels of tRNA^Ile(UAU)^ and tRNA^Ala(AGC)^ in *S. pombe* WT and *pus1*Δ strains at 38.5°C. The statistical significance was evaluated as in Figure 3B. (*C*) Quantification of levels of unspliced pre-tRNA^Ile(UAU)^ and unspliced pre-tRNA^Arg(CCU)^ in *S. pombe* WT and *pus1*Δ strains at 38.5°C. The statistical significance was evaluated as in Figure 3B.

Analysis of tRNA levels shows that a *pus1*Δ *tI(UAU)-i*Δ strain has near normal amounts of tRNA^Ile(UAU)^ after temperature shift from 30°C to 38.5°C in YES media. Whereas the *pus1*Δ *tI(UAU)-WT* has the expected substantially reduced relative levels of tRNA^Ile(UAU)^ after temperature shift, compared to the corresponding *pus1^+^ tI(UAU)-WT* strain (0.27 vs. 1.01), relative tRNA^Ile(UAU)^ levels in the *pus1*Δ *tI(UAU)-i*Δ strain (0.99) are very similar to WT levels, indicating little, if any decay of tRNA^Ile(UAU)^ in this strain (Fig. 6A,B). Comparison with the relative tRNA^Ile(UAU)^ levels at 30°C (Fig. 6A; Supplemental Fig. S10) shows that tRNA^Ile(UAU)^ levels are not simply overproduced in the *pus1*Δ *tI(UAU)-i*Δ strain, supporting the conclusion that tRNA^Ile(UAU)^ does not undergo appreciable decay in the *pus1*Δ *tI(UAU)-i*Δ strain. These results therefore suggest that the decay of tRNA^Ile(UAU)^ in a *pus1*Δ strain is directly due to the presence of its intron.

One plausible mechanism by which tRNA^Ile(UAU)^ decay could be caused by the intron is that unspliced pre-tRNA^Ile(UAU)^ is undergoing the decay. Consistent with this interpretation, we find a substantial increase in the relative amount of unspliced pre-tRNA^Ile(UAU)^ in the *pus1*Δ *tI(UAU)-WT* strain (4.29), which undergoes decay, compared to that in the corresponding *pus1^+^ tI(UAU)-WT* strain (0.85) (Fig. 6A,C). In contrast, there is no obvious accumulation of unspliced pre-tRNA^Arg(CCU)^ in the *pus1*Δ *tI(UAU)-WT* strain. Moreover, analysis at 30°C (Supplemental Fig. S10) also shows substantial accumulation of unspliced pre-tRNA^Ile(UAU)^ in the *pus1*Δ *tI(UAU)-WT* strain, compared to the corresponding *pus1^+^ tI(UAU)-WT* strain (2.42 vs. 1.00), underscoring that the tRNA^Ile(UAU)^ decay that is occurring in a *pus1*Δ strain at 30°C is also associated with pre-tRNA^Ile(UAU)^ accumulation.

Similarly, we find that a *pus1*Δ *tI(UAU)-U27C* strain undergoes little, if any, tRNA^Ile(UAU)^ decay after temperature shift in YES media, as the relative tRNA^Ile(UAU)^ levels after temperature shift are substantially increased (0.60), compared to those in the corresponding *pus1*Δ *tI(UAU)-WT* strain (0.27), and tRNA^Ile(UAU)^ levels are not elevated in the *pus1*Δ *tI(UAU)-U27C* strain at 30°C (Fig. 6A,B; Supplemental Fig. S10). Moreover, the relative levels of unspliced pre-tRNA^Ile(UAU)^ (Fig. 6A,C) are substantially reduced in the *pus1*Δ *tI(UAU)-U27C* strain (0.64), compared to those in the *pus1*Δ *tI(UAU)-WT* strain (4.29) or the corresponding *pus1^+^ tI(UAU)-WT* strain (0.85).

Thus, in a *pus1*Δ strain, the temperature sensitivity caused by the decay of tRNA^Ile(UAU)^ is associated with the accumulation of unspliced pre-tRNA^Ile(UAU)^, and these two phenomena are both efficiently corrected by either elimination of the intron, or stabilization of the anticodon stem with a *tI(UAU)-U27C* variant. From this data, we infer a model in which the temperature sensitivity of a *pus1*Δ strain is due to tRNA^Ile(UAU)^ decay that occurs at the level of pre-tRNA^Ile(UAU)^.

### Intron-containing pre-tRNA^Ile(UAU)^ has a noncanonical predicted structure for tRNA splicing

**FIGURE 7.**
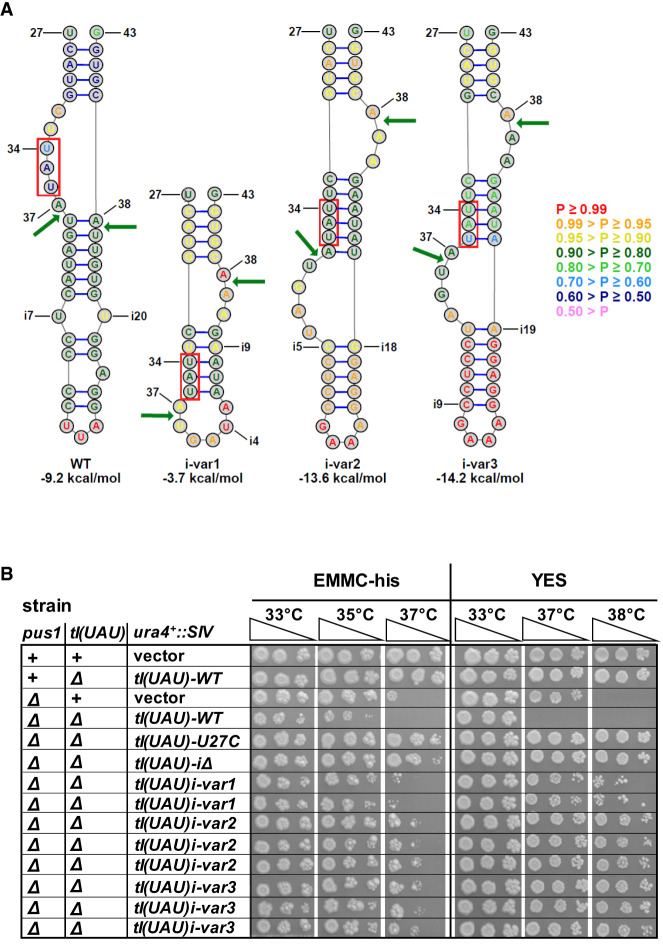
Intron mutations designed to improve pre-tRNA^Ile(UAU)^ structure restore growth in a *pus1*Δ strain. (*A*) A schematic of the ASL-intron region of WT tRNA^Ile(UAU)^, and three variants predicted to have structure compatible with splicing. The schematics indicate nt 27–43 of the tRNA and the intron sequence, which begins one residue after the UAU anticodon (boxed in red). Structures were predicted using the RNAstructure program (https://rna.urmc.rochester.edu/RNAstructureWeb/). Arrows indicate the 5′ and 3′ splice sites that are cleaved by the splicing endonuclease during the first step of splicing. The helix of the BHL motif (i-var1) or the BHB motif (i-var2 and i-var3) is the helix defined by the base pairs involving nt 32 to nt 36 or 37. The legend at the *right* indicates the probability of each base pair. The numbers at the *bottom* represent the predicted folding free energy change (kcal/mol) of the oligomer comprising the ASL-intron region of each variant. (*B*) Growth of *S. pombe pus1*Δ *tI(UAU)-variant* strains. *S. pombe pus1*Δ *tI(UAU)-variant* strains as indicated were grown overnight in YES media at 30°C, and then cells were serially diluted and spotted as described in Figure 1A.

To investigate the cause of the accumulation of unspliced pre-tRNA^Ile(UAU)^ in a *pus1*Δ strain, we examined its likely structure. Multiple biochemical and structural analyses have shown that the tRNA splicing endonuclease optimally recognizes the anticodon stem–loop (ASL) and the intron of unspliced pre-tRNA when they are folded into a bulge-helix-loop (BHL) or bulge-helix-bulge (BHB) secondary structure, in which the 5′ and 3′ splice sites are single-stranded, and the helix is comprised of base pairs between all or part of the anticodon sequence and intron sequences (Baldi et al. 1992; Schmidt and Matera 2020; Hayne et al. 2023a,b; Sekulovski et al. 2023). However, using either of two well-known web servers for secondary structure prediction programs (Mathews et al. 2004; Gruber et al. 2008) (https://rna.urmc.rochester.edu/RNAstructureWeb/; http://rna.tbi.univie.ac.at/), we find that the predicted ASL-intron structure of unmodified pre-tRNA^Ile(UAU)^ has a noncanonical secondary structure, in which the 5′ and 3′ splice sites are not in their optimal BHL or BHB orientation, and are instead directly across from one another, with no helix between the sites (Figs. 4D, 7A). This predicted noncanonical structure is consistent with the accumulation of unspliced pre-tRNA^Ile(UAU)^ that is observed in *pus1*Δ strains and with the model that tRNA^Ile(UAU)^ decay occurs in a *pus1*Δ strain at the level of unspliced pre-tRNA^Ile(UAU)^.

### Intron mutations designed to improve pre-tRNA^Ile(UAU)^ structure restore growth and tRNA levels in a *pus1*Δ strain

To test the model that *pus1*Δ temperature sensitivity is due to decay of unspliced pre-tRNA^Ile(UAU)^, we designed *tI(UAU)* variants bearing intron mutations predicted to improve tRNA splicing efficiency, and then examined the growth properties of a *pus1*Δ strain after replacement of the WT *tI(UAU)* gene with these variants. We designed and tested three such variants, each predicted to have a lowest free energy structure that is compatible with tRNA splicing: a BHL for *tI(UAU)i-var1* with a stability of −3.7 kcal/mol; and BHB structures for *tI(UAU)i-var2* and *tI(UAU)i-var3* with predicted stabilities of −13.6 and −14.2 kcal/mol, respectively (Fig. 7A). Examination of the entire pre-tRNA of each variant results in similar predicted structures, with similar differences in predicted stabilities (Supplemental Fig. S11).

Analysis of the growth properties shows that *pus1*Δ strains with each of these intron variants as the sole source of tRNA^Ile(UAU)^ is demonstrably more temperature resistant than the corresponding *pus1*Δ *tI(UAU)-WT* gene (Fig. 7B). Whereas the *pus1*Δ *tI(UAU)-WT* strain is temperature sensitive in YES media at 37°C and 38°C, the corresponding *pus1*Δ *tI(UAU)i-var1* strain grows well in YES media at 37°C and modestly at 38°C, and the corresponding *pus1*Δ *tI(UAU)i-var2* and *pus1*Δ *tI(UAU)i-var3* strains each grow well in YES media at 37°C and 38°C. Similar results are observed in EMMC-His media.

**FIGURE 8.**
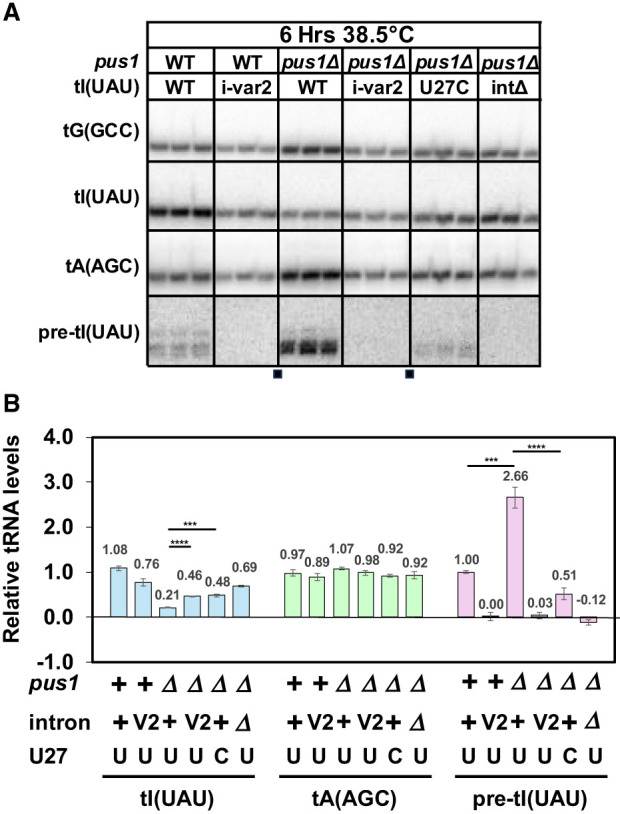
A *pus1*Δ *tI(UAU)i-var2* strain has increased levels of tRNA^Ile(UAU)^ and undergoes little obvious decay of tRNA^Ile(UAU)^. (*A*) Northern analysis of an *S. pombe pus1*Δ *tI(UAU)i-var2* strain after temperature shift. The *S. pombe pus1*Δ *tI(UAU)i-var2* strains and control strains as indicated were grown in YES media at 30°C and shifted to 38.5°C for 6 h, and then RNA was isolated and analyzed by Northern blotting. The image is from a single gel after transfer and hybridization to the indicated probes; small squares at the *bottom* indicate where the images were spliced to remove lanes not shown. (*B*) Quantification of levels of tRNA^Ile(UAU)^, tRNA^Ala(AGC)^, and unspliced pre-tRNA^Ile(UAU)^ in *S. pombe* WT and *pus1*Δ strains at 38.5°C. The statistical significance was evaluated as in Figure 3B.

Analysis of tRNA levels shows that the improved growth of the *pus1*Δ *tI(UAU)i-var2* strain is associated with increased levels of tRNA^Ile(UAU)^ at 38.5°C, accompanied by reduced accumulation of pre-tRNA^Ile(UAU)^ (Fig. 8A,B). Thus, at 38.5°C, the *pus1*Δ *tI(UAU)i-var2* strain has substantially increased relative tRNA^Ile(UAU)^ levels compared to the corresponding *pus1*Δ *tI(UAU)-WT* strain (0.46 vs 0.21), similar to the tRNA^Ile(UAU)^ levels in the *pus1*Δ *tI(UAU)-U27C* strain (0.48). Moreover, in the *pus1*Δ *tI(UAU)-var2* strain, there is a dramatic reduction in the accumulation of unspliced pre-tRNA^Ile(UAU)^ at 38.5°C (0.03), compared to the corresponding *pus1*Δ *tI(UAU)-WT* strain (2.66). Analysis at 30°C (Supplemental Fig. S12) shows that the *pus1*Δ *tI(UAU)i-var2* strain has near normal tRNA^Ile(UAU)^ levels, compared to those in the corresponding *pus1*^*+*^ strain (0.88 vs. 0.85), showing that the increased tRNA^Ile(UAU)^ levels in the *pus1*Δ *tI(UAU)-var2* strain are not due to overexpression of the tRNA.

These results are consistent with the model that a *pus1*Δ strain is temperature sensitive primarily due to decay of unspliced pre-tRNA^Ile(UAU)^, rather than mature tRNA, and suggests an extension of the model, discussed further below, in which the pseudouridine modification of U_34_ and U_36_ triggers splicing of pre-tRNA^Ile(UAU)^.

## DISCUSSION

The results shown here demonstrate that one major role of *S. pombe* Pus1 is to prevent tRNA^Ile(UAU)^ decay by Dhp1 of the RTD pathway. In support of this, we have shown that *S. pombe pus1*Δ strains are temperature sensitive in both rich and minimal media, that this temperature sensitivity is associated with reduced levels of tRNA^Ile(UAU)^, and that overexpression of tRNA^Ile(UAU)^ suppresses the *pus1*Δ temperature sensitivity. Furthermore, among spontaneous suppressors of the *pus1*Δ temperature sensitivity, we find four with *dhp1/rat1* mutations, and two with *tol1/met22* mutations, each of which has increased tRNA^Ile(UAU)^ levels at 38.5°C, with the increase in tRNA^Ile(UAU)^ levels corresponding to the efficiency of suppression. These results argue strongly that *pus1*Δ mutants are temperature sensitive due to decay of tRNA^Ile(UAU)^ by Dhp1/Rat1 of the RTD pathway, with the *tol1/met22* mutations presumed to lead to the accumulation of the metabolite pAp, a known inhibitor of 5′-3′ exonucleases including Rat1/Dhp1 in *S. cerevisiae* (Dichtl et al. 1997; Yun et al. 2018).

Our data showing that tRNA^Ile(UAU)^ decay in *pus1*Δ mutants also occurs at 30°C is part of a general theme in the biology of tRNA body modification mutants affected by RTD, in which the decay is evident in normal growth conditions in which growth is unaffected, but is more severe at higher temperatures, or with an additional modification mutation, or with a drug like 5-FU that inhibits some modifications, resulting in a more pronounced growth defect (Chernyakov et al. 2008; Dewe et al. 2012; Tasak and Phizicky 2022; Bowles and Jackman 2024; De Zoysa et al. 2024; Gorlitz et al. 2024).

Our data are consistent with a model in which *S. pombe pus1*Δ mutants undergo decay of unspliced pre-tRNA^Ile(UAU)^, instead of the expected mechanism involving mature tRNA^Ile(UAU)^ (Chernyakov et al. 2008; Whipple et al. 2011). Three lines of evidence support the model that *S. pombe pus1*Δ mutants undergo decay of pre-tRNA^Ile(UAU)^, rather than mature tRNA^Ile(UAU)^. First, replacement of the sole *tI(UAU)* gene with a *tI(UAU)* gene lacking its intron leads to dramatically increased temperature resistance in the resulting *pus1*Δ *tI(UAU)-i*Δ strain relative to the corresponding *pus1*Δ *tI(UAU)-WT* strain, and no visible decay of tRNA^Ile(UAU)^ at elevated temperature (Figs. 5, 6A,B; Supplemental Fig. S10). Second, a *pus1*Δ *tI(UAU)-WT* strain has distinctly elevated levels of unspliced pre-tRNA^Ile(UAU)^, as would be expected if splicing of pre-tRNA^Ile(UAU)^ is inefficient, allowing decay at this stage (Fig. 6A,C; Supplemental Fig. S10). Third, each of the three *pus1*Δ strains with *tI(UAU)* intron mutations designed to have their ASL-intron region fold into a BHL or BHB structure that is compatible with splicing, improves the temperature resistance of the corresponding *pus1*Δ *tI(UAU)i-var* strains, relative to the *pus1*Δ *tI(UAU)-WT* strain (Fig. 7), and the *pus1*Δ *tI(UAU)i-var2* strain has both increased tRNA^Ile(UAU)^ levels and reduced accumulation of unspliced pre-tRNA^Ile(UAU)^ at high temperature (Fig. 8).

There are two prior reports in *S. cerevisiae* suggesting that tRNA decay occurs at the level of pre-tRNA, in a mechanism that involves Met22. We previously found that decay of a number of fully modified anticodon variants of the tyrosine-inserting nonsense suppressor *SUP4*_*°c*_ occurs at the level of the unspliced pre-tRNA^Tyr^, in a pathway called the Met22-dependent pre-tRNA decay pathway, in which destabilization of the canonical BHL/BHB ASL-intron structure leads to the accumulation of unspliced pre-tRNA (Payea et al. 2020). Recently, the Jackman lab described a novel pathway in which *S. cerevisiae trm10*Δ mutants, lacking m^1^G_9_ in their tRNAs, undergo decay of tRNA^Trp^ (magnified in the presence of 5-FU), accompanied by accumulation of unspliced pre-tRNA^Trp^ (Bowles and Jackman 2024). This decay was inhibited in *met22*Δ mutants but was not inhibited in mutants in any of the known exonucleases that participate in tRNA decay, suggesting a novel pathway. The decay of pre-tRNA^Ile(UAU)^ in *S. pombe pus1*Δ mutants that is described here is similar to these two pathways involving pre-tRNA decay in *S. cerevisiae,* although in this case, Dhp1/Rat1 is clearly involved.

One intriguing aspect of the decay of pre-tRNA^Ile(UAU)^ in *pus1*Δ mutants is the role of the Ψ residues. As the accumulation of unspliced pre-tRNA^Ile(UAU)^ is greatly enhanced in *pus1*Δ mutants compared to WT strains (Figs. 6, 8), a parsimonious explanation is that the pre-tRNA accumulation is caused by the lack of Ψ in the pre-tRNA^Ile(UAU)^, and that the presence of one or more of the Ψ residues drives splicing.

**FIGURE 9.**
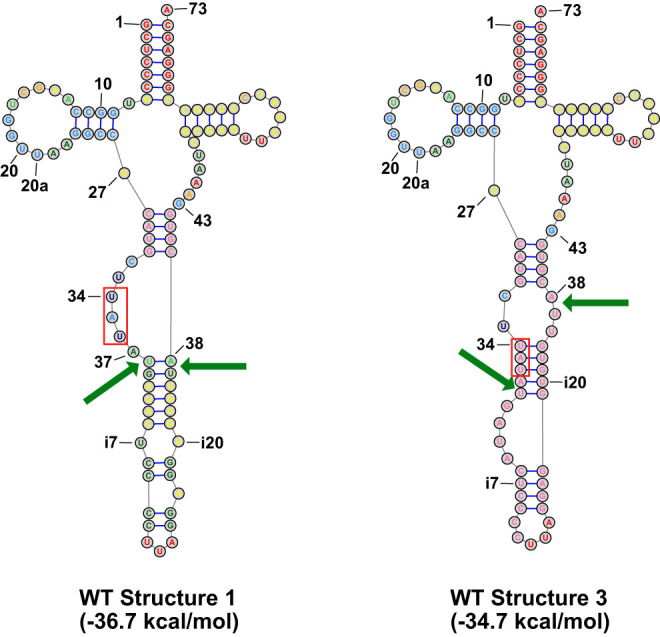
A schematic of two stable predicted conformations of *S. pombe* WT pre-tRNA^Ile(UAU)^ and three variants predicted to have structure compatible with splicing. Structures are shown for the complete unspliced pre-tRNA^Ile(UAU)^, with the UAU anticodon outlined in red, arrows indicating the 5′ and 3′ splice sites, and numbers at the *bottom* represent the predicted folding free energy change (kcal/mol) of the pre-tRNA. The colors of nucleotides are as in Figure 7A, corresponding to the probability of each base pair. Structures were obtained using the RNAstructure program (https://rna.urmc.rochester.edu/RNAstructureWeb/).

Consideration of the predicted structures of pre-tRNA^Ile(UAU)^ suggests a model in which Ψ_34_ and Ψ_36_ modification of pre-tRNA^Ile(UAU)^ stabilizes a pre-tRNA^Ile(UAU)^ structure that is compatible with splicing. Analysis of the spectrum of pre-tRNA^Ile(UAU)^ structures predicted by the RNAstructure Fold program (Bellaousov et al. 2013) shows that the most stable predicted structure (Structure 1) has a stability of −36.7 kcal/mol (Fig. 9), but this structure lacks the canonical BHL/BHB motif that promotes splicing. However, Structure 3 in the set of stable predicted structures is predicted to be only 2 kcal/mol less stable (−34.7 kcal/mol), and this structure has a canonical BHB motif that is compatible with pre-tRNA splicing by the tRNA splicing endonuclease (Schmidt and Matera 2020; Hayne et al. 2023a,b; Sekulovski et al. 2023). Moreover, as pseudouridine substitution of uridine in canonical base pairs is known to stabilize helix formation (Hall and McLaughlin 1991; Meroueh et al. 2000; Sumita et al. 2005; Hudson et al. 2013; Kierzek et al. 2014), it is plausible that this helix of the BHB motif is stabilized when Ψ_34_, and Ψ_36_ are formed in the pre-tRNA^Ile(UAU)^.

Two arguments favor the model that formation of Ψ_34_ and Ψ_36_ on unspliced pre-tRNA^Ile(UAU)^ promotes splicing of the pre-tRNA^Ile(UAU)^, and prevent its decay. First, it seems highly likely that the intron of tRNA^Ile(UAU)^ is required for Ψ_34_ and Ψ_36_ modification by *S. pombe* Pus1, as it is well established in vivo and in vitro that *S. cerevisiae* Pus1 modifies U_34_ and U_36_ of tRNA^Ile(UAU)^ (Simos et al. 1996; Motorin et al. 1998), and that these modifications require the intron (Szweykowska-Kulinska et al. 1994; Hayashi et al. 2019), and it is also known that *S. pombe* and mouse Pus1 proteins have similar specificity for tRNA substrates, including for an *S. cerevisiae* pre-tRNA^Ile(UAU)^ substrate (Chen and Patton 1999; Hellmuth et al. 2000). Second, the stabilization predicted to be imparted by two Ψ residues in the helix of the BHB motif is significant. We can use prior optical melting results to estimate the increased stabilization of the helix shown in Figure 9 (structure 3) that would be imparted by pseudouridylation at specific positions. The Znosko lab (Hudson et al. 2013) reported a set of stacking nearest-neighbor parameters for Ψ-A base pairs in which, on average, the stabilization of Ψ over U per base pair stack is −0.9 ± 0.6 kcal/mol. In separate experiments, the Kierzek lab (Kierzek et al. 2014) reported optical melting measurements of duplexes with Ψ-G base pairs in which, on average, each Ψ-G base pair stack was stabilized by −0.4 ± 0.4 kcal/mol. As the helix in Figure 9 has three stacks involving Ψ-G base pairs, we therefore estimate that the pseudouridylations would increase stability between −1.2 and −2.7 kcal/mol. This increase in predicted stability from the formation of Ψ_34_ and Ψ_36_ in the central helix of the BHB motif of Structure 3 would significantly shift the equilibrium away from Structure 1 and toward Structure 3, thereby promoting splicing.

Based on these arguments, we suggest that pseudouridylation of pre-tRNA^Ile(UAU)^ at U_34_ and U_36_ of the anticodon stabilizes the pre-tRNA for splicing, thereby preventing decay of the pre-tRNA by the 5′-3′ exonuclease Dhp1/Rat1. As a *pus1*Δ strain lacks Ψ_34_ and Ψ_36_, the pre-tRNA^Ile(UAU)^ is longer-lived, resulting in more time available for exonucleolytic attack by Dhp1. The 5′ end of the initial transcript would bear a triphosphate or might be capped (Ohira and Suzuki 2016), making it more resistant to Dhp1/Rat1, but after removal of the 5′ leader and 3′ end formation, the unspliced pre-tRNA^Ile(UAU)^ would have a 5′-monophosphate and would be susceptible to 5′-3′ exonucleases such as Dhp1. Presumably, the pre-tRNA^Ile(UAU)^ lacking Ψ_34_ and Ψ_36_ is exported to the cytoplasm for splicing on the outer surface of the mitochondria, as normally occurs in *S. cerevisiae* and *S. pombe* (Yoshihisa et al. 2003, 2007; Wan and Hopper 2018), but the reduced efficiency of splicing of the pre-tRNA^Ile(UAU)^ would increase the probability of retrograde import of the pre-tRNA^Ile(UAU)^ back to the nucleus (Shaheen and Hopper 2005; Takano et al. 2005) for decay by Dhp1/Rat1, which is known to be nuclear-localized in *S. cerevisiae* and other eukaryotes (Johnson 1997; Nagarajan et al. 2013). Moreover, prior to retrograde nuclear import, the pre-tRNA^Ile(UAU)^, which is not functional for translation (Phizicky and Hopper 2023), would not be sequestered by isoleucyl-tRNA synthetase or EF-1A to keep it in the cytoplasm.

However, an alternative model is that lack of pre-tRNA^Ile(UAU)^ modification of Ψ_34_ and Ψ_36_ prevents export of pre-tRNA^Ile(UAU)^ to the cytoplasm, thereby prolonging its presence in the nucleus and promoting decay by Dhp1. In this case, the proximal cause of the lack of export would be the formation of a noncanonical pre-tRNA^Ile(UAU)^ structure in the absence of Ψ_34_ and Ψ_36_, to prevent export by known exporters like Los1, which binds the tRNA body and the 5′ and 3′ ends (Cook et al. 2009).

In terms of the model in Figure 9, it is intriguing that a U27C variant would efficiently suppress the temperature sensitivity and reduce the accumulation of unspliced pre-tRNA^Ile(UAU)^ that is found in a *pus1*Δ *tI(UAU)-WT* strain, as one might expect that the U27C variant would equally stabilize both Structure 1 and Structure 3. Although this conjecture is true, as each structure is predicted to be stabilized by 2.0 kcal/mol, the substantially more stabilized anticodon stem due to the U27C mutation significantly reduces the population of other possible pre-tRNA structures with relatively similar energy, which would otherwise compete with and reduce the population of structure 3.

Aside from the proposed model in which formation of Ψ_34_ and Ψ_36_ promotes splicing of pre-tRNA^Ile(UAU)^, we also note that the lack of Ψ_34_ and Ψ_36_ affects the function of the mature tRNA^Ile(UAU)^. Thus, a *pus1^+^ tI(UAU)-i*Δ strain, which lacks Ψ_34_ and Ψ_36_, is mildly temperature sensitive on EMMC-His minimal media and to some extent on YES media, compared to the corresponding *pus1^+^ tI(UAU)-WT* strain (Fig. 5). This growth phenotype of the *S. pombe pus1^+^ tI(UAU)-i*Δ strain is plausibly due to reduced efficiency of tRNA^Ile(UAU)^ decoding of AUA codons in the ribosome A site (Grosjean and Westhof 2016); to misdecoding or poor decoding arising from the additional ncm^5^U_34_ modification; or to increased decay of mature tRNA^Ile(UAU)^ lacking Ψ_34_, and Ψ_36_, (perhaps caused by reduced ribosome binding). Similarly, we noted above that in the context of a *tI(UAU)-U27C* variant, the more robust growth of a *pus1^+^ tI(UAU)-U27C* strain than of a *pus1*Δ *tI(UAU)-U27C* strain also suggests significant function of Ψ_34_ and Ψ_36_, as these are the only predicted modification differences in these two strains (Fig. 5A). In *S. cerevisiae*, a slow growth phenotype was also observed under certain growth conditions in strains in which the *tI(UAU)* genes lack their introns, and in this case, it was ruled out that the resulting tRNA^Ile(UAU)^ with ncm^5^U_34_ instead of Ψ_34_, and Ψ_36_ was misdecoding AUG codons (Hayashi et al. 2019).

It remains to be seen if the findings described here extend to other eukaryotic organisms, including *S. cerevisiae* and humans. We note that a large majority of eukaryotic tRNA^Ile(UAU)^ genes have an intron (Chan and Lowe 2016; Schmidt and Matera 2020), and it seems likely that most or all of these will have Ψ_34_ and Ψ_36_, as they do in *S. cerevisiae* and *S. pombe*. It is attractive to speculate that in some eukaryotes, the same circuit is in operation, requiring pseudouridylation for efficient splicing of the pre-tRNA, and Ψ_34_ and Ψ_36_ for efficient function of the mature tRNA. We note that it is not currently understood how U_34_ and U_36_ of pre-tRNA^Ile(UAU)^ are recognized for modification by Pus1, as Pus1 recognizes only a modestly defined structural motif and sequence motif (Sibert and Patton 2012; Carlile et al. 2019), and this may also complicate the pathway for maturation of pre-tRNA^Ile(UAU)^. It is also not clear if the relatively flexible structure of the exon–intron region of the *S. pombe* pre-tRNA^Ile(UAU)^ is a point of regulation in maturation and, if so, if this regulation is conserved in some other eukaryotes. In addition, it also remains to precisely define the roles for Ψ_34_ and Ψ_36_ in mature tRNA^Ile(UAU)^, and to determine the function, if any, of the additional ncm^5^U_34_ modification found in the absence of Ψ_34_ and Ψ_36_.

## MATERIALS AND METHODS

### Yeast strains

*S*. *pombe* strains used in this study are shown in Supplemental Table S1. *S*. *pombe* haploid WT strains were derived from SP286 (*ade6-M210/ade6-M216*, *leu1-32/leu1-32*, *ura4-D18/ura4-D18 h+/h+*) (Kim et al. 2010). *S*. *pombe pus1*Δ::*HygMX* and *pus1*Δ::*kanMX* strains were constructed by Gibson assembly of ∼500 nt 5′ of the *pus1* gene, the drug marker, and ∼500 nt 3′ of the *pus1* gene, followed by linear transformation using lithium acetate (Bahler et al. 1998). Other *S. pombe* deletion strains were constructed similarly. The tI(UAU) variants were integrated at the chromosomal *ura4-D18* locus using a single *ura4*^*+*^ integrating vector containing the corresponding gene under its promoter (Vjestica et al. 2020). All *S. pombe* strains described in this work were made in biological duplicate or triplicate. To reconstruct the *dhp1-S737P* mutation in a WT and *pus1*Δ strain, we first made a plasmid with an insert comprised of nt 1428–3427 of *dhp1* (the 3′ end of the CDS is at nt 2976, and the S737P mutation is at nt 2208), followed by the entire *ura4*^+^ gene, and then nt 2381–3976. After sequencing, this fragment was then transformed into *S. pombe*, selecting for Ura^+^ cells, followed by PCR and sequencing of candidates to identify those in which the mutation was present.

### Plasmids

All plasmids used in this study are listed in Supplemental Table S2. Plasmids expressing *S. pombe* Pus1 and Tol1 were cloned with ∼1000 and 500 bp flanking 5′ and 3′ DNA into a pREP3X-derived plasmid, as described before (De Zoysa and Phizicky 2020; Tasak and Phizicky 2022). Plasmids expressing *S. pombe* tRNA genes were cloned with ∼300 and 100 bp flanking 5′ and 3′ DNA into a pREP3X-derived plasmid.

### *S. pombe* media and growth conditions

*S. pombe* strains were cultivated in rich (YES) or Edinburgh minimal complete media (EMMC) as described (Tasak and Phizicky 2022). For all analysis experiments, strains were grown in biological triplicates in YES or EMMC-Leu media at 30°C to an OD_600_ of 0.4–0.6, and for temperature shift experiments, cells were then diluted to an OD_600_ of 0.1 and grown at 38.5°C for up to 6 h. Cells were harvested at 4°C, washed with cold water, snap-frozen on dry ice, and stored at −80°C.

### Bulk RNA preparation for Northern blot analysis

Bulk RNA was extracted from ∼0.5 to 1.5 OD pellets using acid-washed glass beads and phenol (Elder et al. 1983). The isolated RNA was resolved by polyacrylamide gel electrophoresis (10% PA (19:1), 7 M urea, 1 x TBE) and transferred to a Hybond-N^+^ membrane (Amersham). Then RNAs were analyzed by hybridization to 5′ ^32^P-labeled DNA oligomers (Supplemental Table S3) as described (Alexandrov et al. 2006), followed by imaging on an Amersham Typhoon phosphorimager (Cytiva, Marlborough, MA), and quantification with Image Quant v5.2. tRNA levels were quantified by normalization to the control tRNA^Gly(GCC)^ at that temperature and growth condition, and then to the normalized WT at 30°C (De Zoysa and Phizicky 2020).

### Purification of tRNA for HPLC analysis

*S. pombe* WT and *pus1*Δ strains were cultivated in biological triplicates in YES or EMMC-Leu media at 30°C to mid-log phase, and bulk low molecular weight RNA was extracted from ∼300 OD cell pellets by using hot phenol (Jackman et al. 2003). Canonical-sized tRNAs, comprising the 37 isodecoder tRNA families lacking a long variable loop, were purified from bulk RNA by polyacrylamide gel electrophoresis (8% PA (19:1), 7 M urea, 1 x TBE), followed by phenol extraction of the eluted band, and ethanol precipitation. Specific tRNAs were purified from bulk RNA using streptavidin magnetic beads by hybridization to biotinylated DNA probes (Supplemental Table S4), as described previously (Jackman et al. 2003). To purify tRNA^Ile(UAU)^, *S. pombe* WT and *pus1*Δ strains were first transformed with a [*leu2^+^ tI(UAU)*] plasmid, and transformants were grown in triplicate in EMMC-Leu media to obtain 300 OD cell pellets.

### HPLC analysis of nucleosides of purified tRNA

To analyze nucleosides of purified tRNAs or of canonical-sized tRNAs, 1.25 µg tRNA was treated with P1 nuclease and then with calf intestinal phosphatase (CIP), and the resulting nucleosides were resolved by HPLC at pH 5.0, and quantified by calculating the area under each nucleoside peak at the corresponding maximum absorbance (Jackman et al. 2003). For quantification of the canonical-sized tRNAs, cytidine was used as a reference nucleoside (Tasak and Phizicky 2022).

### CMCT assays

Purified tRNA^Ile(UAU)^ from *S. pombe* WT and *pus1*Δ strains was treated with N-Cyclohexyl-N′-(2-morpholinoethyl)carbodiimide methyl-p-toluenesulfonate (CMCT), followed by alkaline treatment, annealing with 5′ ^32^P-labeled DNA oligos (OFS046 or OFS049), and reverse transcription with AMV polymerase (Promega), as previously described (Huang et al. 2016). Samples were resolved on a 15% polyacrylamide 7 M urea gel and imaged using an Amersham Typhoon phosphorimager.

### Isolation of spontaneous suppressors of the *S. pombe* pus1Δ temperature-sensitive phenotype

*S. pombe pus1*Δ strains derived from multiple independent colonies of the original *pus1*Δ biological triplicates were cultivated in liquid YES media at 30°C overnight, and then 5 × 10^5^, 1 × 10^6^, or 1 × 10^7^ cells were plated onto YES or EMMC-His plates and incubated at 36°C, 37°C, 38°C, or 39°C for several days. A variety of different sizes of spontaneous suppressors were picked and re-streaked onto the same conditions. Single colonies of suppressor strains were then patched onto YES plates and incubated at 30°C, and then overnight cultures in YES media at 30°C were tested again for suppression, and saved.

### Whole-genome sequencing

*S. pombe* strains were cultivated in YES at 30°C. Cells were harvested at 4°C, washed with cold water, snap-frozen on dry ice, and stored at −80°C. Then genomic DNA was extracted from 6 OD pellets using the Quick-DNA Fungal/Bacterial Miniprep Kit (Zymo Research), and libraries were generated using the DNA Prep library prep kit (Illumina). Whole-genome sequencing was performed by the Biotechnology Resource Center of the Cornell University at a read depth of 15–50 X per genome nucleotide using a NextSeq500 (Illumina) instrument.

## SUPPLEMENTAL MATERIAL

Supplemental material is available for this article.

## Figures and Tables

**Franziska Stegemann:**
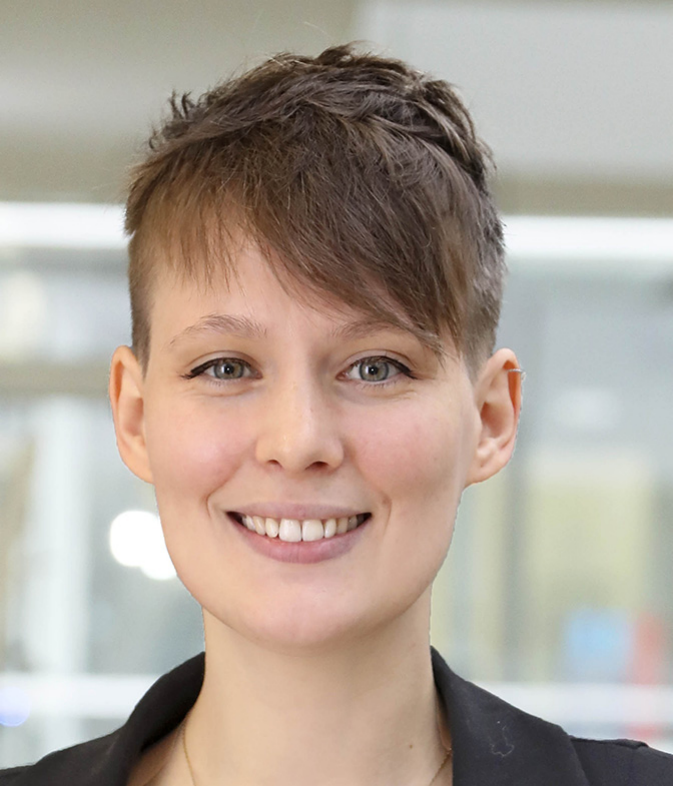


## MEET THE FIRST AUTHOR

***Meet the First Author(s)* is an editorial feature within *RNA*, in which the first author(s) of research-based papers in each issue have the opportunity to introduce themselves and their work to readers of *RNA* and the RNA research community. Franziska *“*Franzi” Stegemann and Erin Marcus are co-first authors of this paper, “*Schizosaccharomyces pombe pus1* mutants are temperature sensitive due to decay of tRNA^Ile(UAU)^ by the 5′-3′ exonuclease Dhp1, primarily targeting the unspliced pre-tRNA.” Franzi was working as a postdoctoral associate in the laboratory of Eric Phizicky in the Department of Biochemistry and Biophysics at the University of Rochester. They were studying the biology of tRNA modifications in *S. pombe* and *S. cerevisiae*.**

**What are the major results described in your paper and how do they impact this branch of the field?**

Our results demonstrate that one major biological role of *S. pombe* Pus1 is to modify U_27_, U_34_, and U_36_ of tRNA^Ile(UAU)^, preventing decay of unspliced pre-tRNA^Ile(UAU)^ by the 5′-3′ exonuclease Dhp1, implying a substantial role for one or more of the Ψ residues in stabilizing the pre-tRNA structure for splicing. Our findings provide new insights into the regulation of tRNA maturation and quality control pathways, expanding our knowledge of tRNA biology in eukaryotes.

**What led you to study RNA or this aspect of RNA science?**

My fascination with complex biological pathways began during my undergraduate studies, where I was first exposed to the intricacies of biological systems. During my doctoral research on enzyme kinetics, my curiosity deepened, driving me to explore broader biological complexities. This led me to RNA biology, where I became particularly interested in the logic and complexity of RNA quality control pathways in eukaryotes. I focused on the biology of tRNA modifications and tRNA decay pathways, as these processes are crucial for understanding fundamental biology and diseases linked to tRNA dysregulation.

**What are some of the landmark moments that provoked your interest in science or your development as a scientist?**

My interest in science began as a child, fueled by a natural curiosity and a desire to understand the world around me—passions that were nurtured by my parents. In school, I excelled in chemistry, biology, and physics, which deepened my fascination with how things work. I developed a particular love for logic and problem-solving, which has guided my scientific journey ever since. Throughout my undergraduate, graduate, and postdoctoral studies, I encountered many inspiring role models who further solidified my commitment to science. These experiences laid the foundation for my career and continue to fuel my drive to explore complex biological systems.

**If you were able to give one piece of advice to your younger self, what would that be?**

Early on, I often felt pressure to have everything figured out, but I've since learned that progress comes with patience and persistence. It's okay to not have all the answers right away. Stay curious, be open to new ideas, and embrace the challenges that come your way—they're opportunities for growth. Most importantly, don't be afraid to take risks, because stepping outside your comfort zone often leads to real breakthroughs.

**What were the strongest aspects of your collaboration as co-first authors?**

The strongest aspect of our collaboration as co-first authors was the team-oriented approach we took, with each of us contributing our unique strengths while working closely with a supportive group of colleagues. We fostered an environment of open communication and collaboration, where ideas were freely exchanged, and challenges were tackled collectively. The shared passion for the research topic, combined with the team's collective expertise, ensured that the project progressed smoothly and that every aspect of the research was carefully refined. It was this collaborative spirit that truly drove the success of our work.
